# Derivation of indoor air guidance values for volatile organic compounds (VOC) emitted from polyurethane flexible foam: VOC with repeated dose toxicity data

**DOI:** 10.17179/excli2018-1440

**Published:** 2018-08-07

**Authors:** Thomas Schupp

**Affiliations:** 1 Muenster University of Applied Science, Chemical Engineering, Stegerwaldstrasse 39, D-48565 Steinfurt, GERMANY

**Keywords:** indoor air, guidance values, consumer exposure, risk, chamber test

## Abstract

Polyurethane Flexible Foams (PUF) are versatile materials used in upholstered furniture and bed mattresses. Due to the production procedure, fresh foams emit volatile organic compounds (VOC). Chamber tests issued by the European association of flexible polyurethane foam blocks manufacturers (EUROPUR) revealed certain levels of VOCs, and the emission rates are declining over time. To assess the risk associated to these VOCs which, as a consequence, might be detectable in indoor air where these PUF are used. To evaluate the risk for consumers, their concentration can be matched against existing benchmarks for indoor air. These benchmarks are, for example, guidance values derived by the Advisory Group for Indoor Air Guidance Values of the German UBA (RW-values), Lowest Concentrations of Interest (LCI) for building products, or against derived no effect levels (DNELs) for consumers, defined in registration dossiers under the European Regulation No. 1907/2006. In this paper, indoor air guidance values are derived for some VOCs which do have neither RW- nor LCI-values, and no DNELs for consumers. Substances covered are trimethylsilanol, fluorotrimethylsilane, chloropropanol, propanal, triethylenediamine and 2,2,3,3-tetramethyl succinodinitrile.

## Introduction

In the late 1990's, the European association of flexible polyurethane foam blocks manufacturers (EUROPUR) started to perform VOC emission tests for flexible polyurethane foams. This activity was introduced as part of a responsible care program to identify potentials for product improvement, and to address any health concerns consumers may have with regards to volatile organic compounds (VOC) from articles containing polyurethane flexible foam (PUF). Selected examples of different PUF types were submitted to emission testing according to ISO 16000. Measurement results and a toxicological evaluation were published by Hillier et al. (2003[13]). Based on these experiences, VOC emission testing in chamber tests became part of EUROPURs product label CERTIPUR^®^ (EUROPUR CERTIPUR, 2018[12]). Since 2003, many additional data concerning foam emissions were generated. Due to the European Union Regulation (EC) 1907/2006, more toxicological data for substances have become available, and producers and importers of chemicals are asked to define Derived No Effect Levels (DNELs) for consumers, if they may be exposed. Further, due to technical development chemicals available on the market and their quality may have changed. EUROPUR decided to issue an update of the Hillier et al. (2003[13]) publication. Concerning the toxicological evaluation of emitted VOCs, several approaches are possible. In 2012, the Advisory Group for Indoor Air Guidance Values of the German UBA (Ausschuss für Innenraumrichtwerte des Umweltbundesamtes) published a revised guidance for the derivation of indoor air guidance values I and II (RW I, RW II). RW I is the precautionary value and is based upon NOAEC, NOAEL or BMDL5 as point of departure. It describes a concentration in indoor air that is not expected to pose a critical health risk to residents. RW II uses the LOAEC, LOAEL or BMDL_10_ as point of departure and describes an indoor air concentration that requests immediate remediation action if exceeded (UBA, 2012[22]). The concept of “Lowest Concentration of Interest” (LCI) addresses the testing of building products. LCI values are those concentrations for VOCs in chamber tests that must not be exceeded if the construction product shall be classified as fit for the market. The Joint Research Center points out that the LCI values are to be used to evaluate construction products, not indoor air (Joint Research Center, 2013[17]). However, because the test conditions for the building products simulate later standard indoor situations, and because the LCI is derived quite similar to the “Derived No Effect Level” (DNEL) for consumers under the REACH regulation (ECHA, 2012[11]), existing LCI values may be regarded as equivalent to the DNELs for consumers. Both, the LCI and the DNEL derivation, make use of NOAECs or NOAELs or BMDL_5_.

For some of the VOCs identified in EUROPURs chamber tests, benchmarks for tolerable indoor air concentrations were not available. In this paper, indoor air guidance values are derived for those VOCs which have at minimum repeated dose toxicity data, or where due to instability in an aqueous environment the rapid formation of hydrolysis products is expected, and these hydrolysis products have the required minimum data set.

For the derivation of an Indoor Air Guidance Value (IAGV), the following data set is required: a) repeated dose studies (preferably by inhalation exposure); b) a chronic cancer study unless mutagenicity studies indicate no genotoxic risk (at least data from the bacterial reverse mutation assay, point mutation and chromosomal aberration in mammalian cells); c) reproduction toxicity studies addressing development and fertility (at least screening studies); the endpoint fertility may be addressed by histopathology of reproductive organs in subchronic or chronic studies. Local, systemic and reproduction effects are addressed separately (see Table 1(Tab. 1)). The reasons for this differentiation are different extrapolation factors and different data density and data quality of the respective data bases. For example, concerning systemic toxicity the data base may be sufficient to derive and IAGV_systemic_, but for reproduction effects the data base may be limited and, therefore, the IAGV is called tentative IAGV (TIAGV_repro_) only.

## Methods

Substances identified in chamber tests without RW I, LCI or DNEL values were evaluated similar to the RW concept methodology (UBA, 2012[22]). If appropriate, Indoor Air Guidance Values (IAGVs) are derived for systemic and local effects separately. The Points of Departure (PoD) are typically animal data, but available human data have to be taken into account. Generally, only repeated dose inhalation studies are suitable for the derivation of an IAGV.

In case of absence of repeated dose inhalation studies, all three concepts allow to make use of repeated dose oral studies, provided there is no indication - either by existing toxicity data or by structural alerts - that target organs and severity of effects are likely to be route-dependent. As a default, it is assumed that an orally applied substance is resorbed by 50 %, whereas inhalation exposure is assumed to result in 100 % resorption. For the oral to inhalation exposure, according to the REACH Guidance (ECHA, 2012[11]), 60 kg body weight and a daily breathing volume of 20 m³ are default values for the consumer. For the time extrapolation, all three schemes - RW, LCI, DNEL - make use of default values 6 for subacute to chronic extrapolation, and 2 for subchronic to chronic extrapolation. In the RW concept, time scaling is to be applied to convert intermittent exposure in animal studies, p. e. 5 d per week and 6 h per day, to permanent exposure conditions, which are 7 d per week and 24 h per day (168 h per week). For the inter-species extrapolation, toxicokinetic differences are to be covered by allometric scaling if oral data on an mg/kg body weight basis are used. 

Remaining inter-species differences are covered by a default factor (toxicodynamic factor) of 2.5. For local effects after inhalation, the RW-concept of the UBA recommends a factor of 1 for physical-chemical irritation; for systemic effects this factor is also 1 if the most sensitive of at least two studies was used as point of departure; a factor of 2.5 is used if only one study is available and metabolism is likely to play a role in detoxificition (UBA, 2012[22]). The RW concept does not explicitly address the severity of effects (UBA, 2012[22]). Carcinogens, however, are evaluated in that respect that the concentration of the carcinogen that is associated with an additional carcinogenic of 10^-6^ shall be established, and measured indoor air concentrations shall be matched against this value (UBA, 2015[21]). 

For the derivation of indoor air guidance values (IAGV) for VOC without RW-, LCI-values or DNELs, extrapolation factors used in this work are summarized in Table 1(Tab. 1).

The IAGV can be defined for systemic, local, and reproduction effects. An IAGV_local_ is derived if repeated dose inhalation studies are available with an LOAEC for local effects. An IAGV is called tentative (TIAGV) under the following circumstances: a) the data base is incomplete (p. e. older studies which do not exactly match todays standards of the OECD test guidelines); b) in case of conflicting results between equivalent studies which can not be resolved yet; c) when it is debatable whether an effect is a NOAEC/NOAEL or LOAEC/ LOAEL.

## Results

A detailed derivation of indoor air guidance values for the VOCs is provided in supplementary material. The following sections summarize the relevant information and results.

### 1-Chloro-2-propanol (CP)

Chloropropanol is genotoxic, but did not induce significant increase in tumors in drinking water studies with rats and mice (NTP, 1998[19]). In a one generation study with rats, CP did not affect fertility and development (ACGIH, 2002[1]); for that reason, the factor to cover children is set to 1. In a subacute inhalation study with rats, a NOAEC of 30 ppm could be established (ACGIH, 2002[1]). Based on available data, the ACGIH (2002[1]) derived an occupational exposure limit of 1 ppm (3.78 mg/m³). The NOAEC of the subacute rat study will be taken as PoD for the derivation of the Indoor Air Guidance Value (IAGV): 

IAGV_CP_: 0.05 ppm = 195 µg/m³.

If the OEL of 1 ppm was taken as point of departure, factors (divisors) of 2 for worker to consumer, 4.2 for 24 h/d and 7 d/w exposure, and a factor of 2 for children would result in an IAGV of 0.06 ppm.

### 2,2,3,3-Tetramethylsuccinodinitrile 

Tetramethylsuccinodinitrile (TMSD) was negative in in-vitro mutagenicity tests (Seifried et al., 2006[20]). Data concerning effects on development are very limited (Doherty et al., 1983[7]), and concerning fertility it is not clear whether or not histopathology of gonads was evaluated in subchronic studies (Johannsen and Levinskas, 1986[16]). In subchronic oral studies with rats and dogs, the NOAEL was 1 mg/kg b.w., which serves as a starting point; in both species, 3 mg/kg was the LOAEC (DFG, 2001[6]). Both subchronic studies, as well as acute toxicity studies indicate that inter-species differences are very small to negligible. For that reason, a divisor of 1.4 is introduced for the allometric scaling from dog to man. Differences in toxicodynamics are regarded as negligible. For intra-species extrapolation, a factor of 10 is used for the consumer. For subchronic to chronic exposure, the factor applied is 2, and a factor 2 addresses children as vulnerable population. Acute toxicity data have demonstrated that orally applied TMSD is completely resorbed; therefore, the oral to inhalation extrapolation factor is 1. Based on the available data, the Tentative Indoor Air Guidance Value is

TIAGV: 54 µg/m³.

Due to open questions / data gaps concerning reproduction toxicity, the indoor air guidance value is regarded as tentative.

### Triethylenediamine 

Triethylenediamine was registered under Regulation (EU) 1907/2006 (REACH), but the registrant did not derive a DNEL for inhalation exposure of the consumer (ECHA Triethylenediamine, 2017[10]). The registrant regards 620 mg/m³ to be the systemic NOAEC in the subacute inhalation study. However, at this concentration, absolute and relative testicle weights, adrenal weights, blood urea and aspartate amino transferase were elevated, and food intake was reduced in male rats. Histopathology did not reveal any adverse effects at the top concentration, but urine analysis was not performed, and tests concerning fertility effects on male rats after inhalation exposure are missing. After oral exposure, triethylenediamine did affect neither fertility nor development in a screening study with rats (ECHA Triethylenediamine, 2017[10]). As a precautionary measure, 63 mg/m³ are regarded as the appropriate point of departure for the derivation of a tentative Indoor Air Guidance Value, which is

TIAGV_systemic_: 38 µg/m³.

If the NOAEL of the reproduction toxicity screening study is taken as point of departure, against the endpoint developmental toxicity and fertility a factor of 5 needs to be introduced due to limited sensitivity of screening studies. The extrapolated NOAEL than is 60 mg/kg/d. A factor of 10 for each, inter- and intra-species extrapolation would result in a NOAEL_man_ of 0.6 mg/kg/d. For a person of 60 kg body weight and 20 m³ breathing volume per day, with a correction factor of 2 (divisor) for oral to inhalation extrapolation results in

IAGV_repro_: 900 µg/m³.

Concerning local effects, the NOAEC_local_ was 5.8 mg/m³ in the subacute inhalation study. On this basis, the Tentative Indoor Air Guidance Value is 

IAGV_local_: 7 µg/m³. 

### Propanal

For propanal (propionaldehyde), some *in vitro* tests indicate genotoxicity whereas others were negative. The sole *in vivo* study identified indicates some weak clastogenic activity in bone marrow of male mice. For acetaldehyde, the MAK-Commission of the Deutsche Forschungsgemeinschaft has drawn the conclusion that it is genotoxic, but irritation is expected to be a requisite for tumor development in chronically exposed rats; therefore, 50 ppm, the subacute NOAEC from rat inhalation studies, devided by a factor of 3, is proposed as MAK-value for acetaldehyde (DFG, 2013[3]). 150 ppm is the LOEC concerning vacuolization of the olfactoric epithelium for propanal. Irritating potencies are comparable between propanal and acetaldehyde, acetaldehyde being a slightly stronger respiratory irritant; acute RD_50_ values in rats are about 3000 ppm for acetaldehyde and 6800 ppm for propanal (Babiuk et al., 1985[2]). 150 ppm will be taken as a NOAEC for systemic, and as LOEC for local toxicity of propanal. The genotoxic potential of propanal may need further clarification. Concerning reproduction toxicity, data from a rat screening study with inhalation exposure are available (ECHA Propanal, 2017[9]). 

IAGV_propanal,systemic_: 300 µg/m³

IAGV_propanal,local_: 200 µg/m³.

For reproduction toxicity, the result is

TIAGV_propanal,repro_: 7.11 mg/m³.

### Trimethylsilanol 

For the derivation of a tentative indoor air guidance level for systemic effects, only the summary of an oral subacute study in rats is available; however, this study was summarized by a governmental body (NIHS, 2017[18]) and, therefore, is regarded as sufficiently reliable. Trimethylsilanol (TMSOH) does not induce point mutations *in vitro*, but there are indications of clastogenic activity; however, this activity was not detectable in *in vivo* assays (Isquith et al*.*, 1988[14][15]). Carcinogenicity data are available for hexamethyldisiloxane (HMDS), which hydrolyses to TMSOH; because of this, data are read across from this compound to TMSOH. Hexamethyldisiloxane does not pose a human health relevant carcinogenic risk after inhalation exposure; the increase in Leydig cell tumors in rats was attributed to a promoting effect of the substance, as this tumor type had a high background rate already (ECHA Hydroxytrimethylsilane, 2017[8]). TMSOH did not affect male fertility in a dominant lethal test with rats (Isquith et al., 1988[15]). In a two-generation study with hexamethyldisiloxane given via inhalation to rats, fertility was not affected; 400 ppm was the NOAEC, higher concentrations caused liver effects in F0 animals (ECHA Hydroxytrimethylsilane, 2017[8]).

The NOAEL from the subacute gavage study in rats is taken as point of departure and extrapolated by several divisors to generate the tentative indoor air guidance value of

TIAGV_systemic_: 195 µg/m³.

The structural analogue hexamethyldisiloxane (HMDS), which is in equilibrium with TMSOH in aqueous solutions, allows to derive a TIAGV for local effects. In a chronic study, 400 ppm HMDS (2750 mg/m³) was the local NOAEC; higher concentrations caused eosinophilic infiltration in respiratory and olfactoric mucosa (ECHA Hexamethyldisiloxane, 2017[8]). The tentative indoor air guidance value for local effects, via this read across, is

TIAGV_local_: 19.6 mg/m³

### Fluoro(trimethyl)silane

Fluoro(trimethyl)silane (FTMS) has a very limited toxicological database, but due to its chemical properties it is expected to hydrolyse rapidly in biological tissues. On hydrolysis, trimthylsilanol (TMSOH) and hydrogen fluoride are formed. Therefore, the tentative indoor air guidance value will be based on these hydrolysis products. 

For hydrogen fluoride, the evaluation of the MAK Commission of the Deutsche Forschungsgemeinschaft was used for the derivation of an Indoor Air Guidance Values (IAGV) (DFG, 2001[4], 2006[5]). For workers, the occupational exposure limit (OEL) was 2 ppm for local, and 1 ppm for systemic effects (DFG, 2001[4], 2006[5]). On this basis, by assuming a factor 2 higher sensitivity of the general population and a factor of 4.2 extended exposure period per week, the results for hydrogen fluoride are

IAGV_HF,systemic_: 0.06 ppm

IAGV_HF, local_: 0.12 ppm.

Every molecule FTMS hydrolyses to one molecule TMSOH and one molecule HF. The tentative indoor air guidance value for FTMS, therefore, is calculated as the result of the two hydrolysis products:


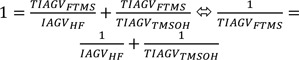


TIAGV_FTMS, local_: 0.12 ppm = 442 µg/m³

TIAGV_FTMS,systemic_: 0.028 ppm = 107 µg/m³

## Discussion

Indoor Air Guidance Values (IAGV) have been derived for those VOCs emitted from polyurethane flexible foam, which up to now have no RW-values (UBA, 2012[22]), LCI-values (Joint Research Center, 2013[17]) or DNEL for consumer (ECHA, 2012[11]). 

Tables 2(Tab. 2) and 3(Tab. 3) summarize the Indoor Air Guidance Values for systemic and local effects. 

Data gaps or remaining uncertainties resulted in the declaration of “tentative” indoor air guidance values. For tetramethylsuccinodinitril, triethylenediamine and propanal, the potential for reproduction toxic effects needs further clarification. It has to be noted, that absence of adverse effects in reproduction toxicity screening studies is not taken as convincing evidence for absence of any adverse effects to reproduction (ECHA, 2012[11]). For trimethylsilanol, only the abstract of a subacute study in rat with oral dosing was available. However, as hexamethyldisiloxane (HMDS) is in equilibrium with trimethylsilanol in aqueous solutions, data from HMDS were used to support the derivation of an TIAGV. For fluoro(trimethyl)silane, the toxicological data base is limited to a few acute data. Due to its instability in aqueous solutions, a TIAGV was derived on the basis of its hydrolysis products.

Indoor Air Guidance Values are derived on a toxicological data basis and are deemed to be protective for the average general population. However, such schemes as presented here can not guarantee absolute safety due to the inter-individual differences in personal susceptibility. That is, although (T)IAGVs are met, severe adverse responses of susceptible individuals can not be excluded with 100 % certainty. With the exception of chloropropanol, the derived indoor air guidance values are regarded as tentative due to limited data - namely against reproduction toxicity - or because of remaining uncertainties, p. e. mutagenicity for propanal.

## Acknowledgements

This research was funded by EUROPUR. The views and interpretations presented are those of the author and not necessarily those of EUROPUR.

## Declaration

The author worked until 2012 for BASF, a major producer of polyurethane flexible foam raw materials.

## Supplementary Material

Supplementary material

## Figures and Tables

**Table 1:**
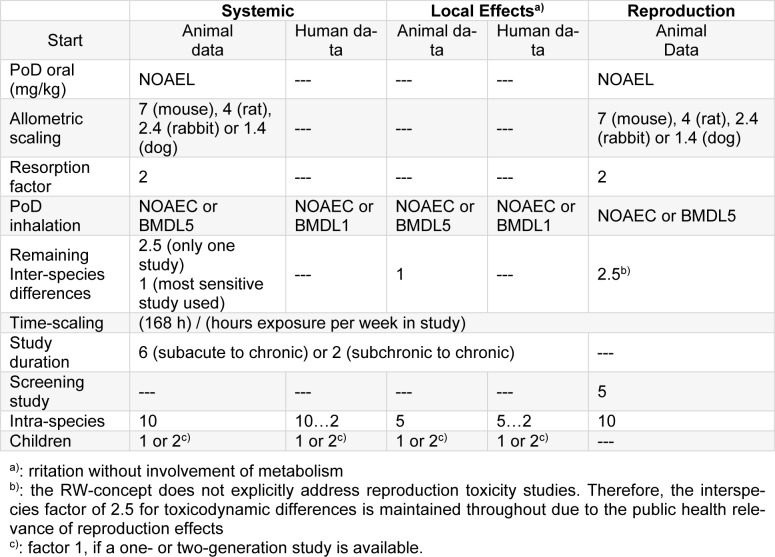
Default extrapolation factors for (tentative) indoor air guidance values, (T)IAGV

**Table 2:**
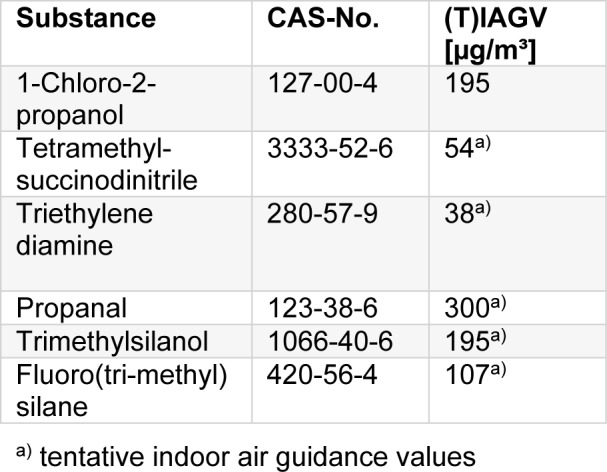
IAGV for systemic effects

**Table 3:**
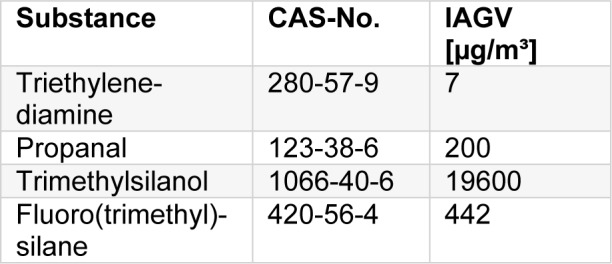
IAGV for local effects
