# Supplementary material for: Derivation of indoor air guidance values for volatile organic compounds (VOC) emitted from polyurethane flexible foam: VOC with repeated dose toxicity data
Source: EXCLI J. 2018 Aug 7;17:784–91. doi: 10.17179/excli2018-1440 (PMC6123614; doi:10.17179/excli2018-1440)
Supplement: Supplementary material [file EXCLI-17-784-s-001.pdf]

EXCLI Journal 2018;17:784-791 – ISSN 1611-2156
Received: June 18, 2018, accepted: August 02, 2018, published: August 07, 2018

Supplementary material to:

DERIVATION OF INDOOR AIR GUIDANCE VALUES FOR VOLATILE ORGANIC COMPOUNDS (VOC) EMITTED FROM POLYURETHANE FLEXIBLE FOAM: VOC WITH REPEATED DOSE TOXICITY DATA

Thomas Schupp

Muenster University of Applied Science, Chemical Engineering, Stegerwaldstrasse 39, D-48565 Steinfurt, GERMANY, Tel.: +49 2551 962595;
E-mail: thomas.schupp@fh-muenster.de

http://dx.doi.org/10.17179/excli2018-1440

This is an Open Access article distributed under the terms of the Creative Commons Attribution License (http://creativecommons.org/licenses/by/4.0/).

Detailed synopsis of the derivation of Indoor Air Guidance Values

TRIMETHYLSILANOL

Identification, physical properties

Name of substance, abbreviation:

→ Trimethylsilanol
→ Hydroxytrimethylsilane
→ TMSOH

CAS-No.: 1066-40-6
Molecular weight: 90.2 g/mol

Table A1: Physical chemical data

Melting point	-12 °C
Boiling point	98 °C
Water solubility	0.995 g/L at 25 °C
Vapor pressure	2900 Pa at 23 °C
Log Pow	1.62 at 20 °C

(GESTIS, 2017c).

Conversion factors air-concentration:
1 ppm = 3.75 mg/m³;
1 mg/m³ = 0.267 ppm

Structure:

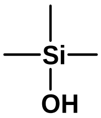

Adsorption, distribution, metabolism, elimination

There are no data available on this substance. Trimethylsilanol (TMSOH) is not stable in aqueous solution but undergoes a condensation reaction to hexamethyldisiloxane (HMDS):

$$2\ (CH_3)_3Si\text{-}OH = (CH_3)_3Si\text{-}O\text{-}Si(CH_3)_3 + H_2O;$$

$$\frac{[HMDS]*[H_2O]}{[TMSOH]^2} = K = 130;$$

The reaction is catalyzed by acid; second order rate constant in dioxane, devided by the acid concentration, is 0.25 L²/mol²/s; in case of base catalysis, the rate constant divided by the NaOH concentration is 0.01 L/mol/s (Rutz et al., 1985). With the latter rate constant, at 1 mol/L as start concentration, the half-life is about 100 seconds; at 1 mmol/L, the half-life increases to about 28 h. For the hydrolysis of hexamethyldisiloxane in aqueous solution, the half-life is achieved after about 5 days (ECHA, 2017b). In an NMR-study, equilibrium constants for the condensation of K = 49…76 were identified (Sefcik et al., 1999). In equilibrium in aqueous solution, about 35…60 % of the material is present as hexamethyldisiloxane, if the concentration of water is set to 55.56 mol/L.

Short-term toxicity

LD_{50}/oral/rat: 2800 mg/kg (Sigma-Aldrich, 2014). Harmful if inhaled (ECHA Hydroxytrimethylsilane, 2017). Not irritating to the skin (Sigma-Aldrich, 2014).

Repeated dose toxicity

Authorities of Japan cite an OECD 407 subacute study (NIHS, 2017):

"Trimethylsilanol was studied for oral toxicity in rats in a 28-day repeat dose toxicity test at doses of 0, 10, 40, 160 and 640 mg/kg. Decreased spontaneous motor activity and staggering gait were observed in both sexes given 640 mg/kg. Moreover, gait difficulty was observed in males given 640 mg/kg. Body weight gain tended to be suppressed and fibrinogen increased in both sexes given 640 mg/kg, along with absolute thymus decrease and relative liver weight increase in females. The changes in body weights in males and in relative liver weights in females were still found at the end of a 14-day recovery period. The NOEL for repeat dose toxicity is considered to be 160 mg/kg/day for both sexes." The substance was dosed in corn oil via gavage.

Genetic toxicity

Trimethylsilanol was not mutagenic in *Salmonella typhimurium* TA100, TA1535, TA98 and TA1537 or *Escherichia coli* WP2 uvrA with and without metabolic activation. (NIHS, 2017; Isquith et al., 1988a).

"Trimethylsilanol did not induce structural chromosomal aberrations or polyploidy in CHL/IU cells with or without an exogenous metabolic activation system." (NIHS, 2017).

In mouse lymphoma cells, TMSOH did not induce point mutations, but sister chromatid exchanges in absence of metabolic activation and chromosomal aberrations in absence and presence of metabolic activation (Isquith et al., 1988a).

TMSOH in DMSO was dosed intraperitoneal in rats. After selected time points, bone marrow was processed for the investigation of structural chromosomal damage. The substance was slightly positive at 48 h harvesting time concerning chromatid gaps; however, the authors rated this finding as not relevant because the number of gaps was still within the historical control, and controls in the study had an unusual low incidence for gaps; more complex chromosomal damages were not elevated. When the test was repeated, there was no statistical significant increase in chromosome damage (Isquith et al., 1988b).

For a dominant lethal test, male rats received TMSOH at 0, 20, 100 or 200 mg/kg body weight per gavage 5 times per week over 8 weeks; in week 9, they were mated with female rats for 5 days per week over 2 weeks. Two weeks after mating, female rats were investigated for corpora lutea, dead and living implants. The substance did not cause an increase in pre-implantation losses or foetal resorptions (Isquith et al., 1988b).

In the *in vivo* studies, no data are reported that allow to judge whether or not the target tissue was reached by the substance. However, as TMSOH is a small molecule with appreciable water solubility and a medium Log Kow, i. p. injection is expected to result in bone marrow exposure. Taken together, the genotoxicity of TMSOH is deemed to be negligible.

Carcinogenicity

There are no data available for TMSOH. Due to the very low to negligible genotoxicity, TMSOH is not expected to pose a carcinogenic risk at dosages below the thresholds for systemic toxicity.

Hexamethyldisiloxane (HMDS) in aqueous solutions is at equilibrium with TMSOH. Rats were exposed against 0, 100, 400, 1600 or 5000 ppm (0, 0.7, 2.75, 10.5 or 32 mg/L) hexamethyldisiloxane (HMDS) for 6 h/d, 5 d/w for 24 months (ECHA Hexamethyldisiloxane, 2017). The top dose was the NOAEC concerning carcinogenicity. 100 ppm was the NOAEC in male rats as higher concentrations caused nephropathy. Male rats had elevated incidences of Leydig cell tumors, but the authors suggest that these findings were attributable to a promoting effect of HMDS, as the background incidence of this tumor type was considerable already. For local effects in lung and upper respiratory tract, the NOAEC was 400 ppm (2750 mg/m³); higher concentrations caused eosinophilic infiltration in the respiratory and olfactoric epithelium. The authors considered effects in the kidneys as species-specific (ECHA Hexamethyldisiloxane, 2017); for systemic effects, the human-relevant NOAEC was 5000 ppm. These data generated with HMDS support the view that TMSOH is unlikely to pose a significant carcinogenic risk.

Reproduction toxicity

In a dominant lethal assay, TMSOH did not affect the fertility index of male rats up to the top dose level of 200 mg/kg/d (5 d/w, 5 w) (Isquith et al., 1988b).

In a two generation study, rats were exposed against 0, 100, 400, 1600 or 5000 ppm HMDS 6 h/d and 7 d/w (ECHA Hexamethyldisiloxane, 2017). Based on periportal pigmentation and increased liver weights, 400 ppm (2750 mg/m³) was the NOAEC in F1 animals. Fertility was not affected. 1600 ppm was the NOAEC for neonatal toxicity due to reduced body weights at 5000 ppm. Concerning teratogenicity, the NOAEC was 5000 ppm.

EXCLI Journal 2018;17:784-791 – ISSN 1611-2156
Received: June 18, 2018, accepted: August 02, 2018, published: August 07, 2018

Indoor Air Guidance Value

For the derivation of a tentative indoor air guidance level, only the summary of an oral subacute study in rats is available; however, this study was summarized by a governmental body (NIHS, 2017) and, therefore, is regarded as reliable. TMSOH does not induce point mutations *in vitro*, but there are indications of weak clastogenic activity *in vitro*; however, this activity was not detectable in *in vivo* assays (Isquith et al., 1988a, b). Carcinogenicity data are available for hexamethyldisiloxane, which hydrolyses to TMSOH; because of this, data are read across from this compound to TMSOH. Hexamethyldisiloxane does not pose a human health relevant carcinogenic risk after inhalation exposure; the increase in Leydig cell tumors in rats was attributed to a promoting effect of the substance, as this tumor type had a high background rate already (ECHA Hexamethyldisiloxane, 2017). TMSOH did not affect male fertility in a dominant lethal test with rats (Isquith et al., 1988b). In a two-generation study with HMDS given via inhalation to rats, fertility was not affected; 400 ppm was the NOAEC, higher concentrations caused liver effects in F0 animals (ECHA Hexamethyldisiloxane, 2017).

The NOAEL from the subacute gavage study in rats is taken as point of departure and extrapolated by several divisors:

$NOAEL_{rat,}$	160 mg/kg/d,
subacute to chronic	6,
inter-species	10,
intra-species	10,
children	2,
$DNEL_{consumer, oral}$	130 µg/kg/d,
oral to inhalation	2,

and for a person with 60 kg bodyweight and 20 m³ breathing volume per day:

$TIAGV_{systemic}$	95 µg/m³.

This TIAGV is expected to be protective concerning effects on male fertility. Data from a two-generation study with the structural analogue hexamethyldisiloxane do not indicate a significant risk to reproduction.

The structural analogue hexamethyldisiloxane, which is in equilibrium with TMSOH in aqueous solutions, allows to derive a TIAGV for local effects. In a chronic study, 400 ppm (2750 mg/m³) was the local NOAEC; higher concentrations caused eosinophilic infiltration in respiratory and olfactoric mucosa (ECHA Hexamethyldisiloxane, 2017).

$NOAEC_{rat, local, chronic}$	2750 mg/m³,
time scaling	: (24/6 x 7/5,
inter-species	: 2.5,
intra-species	: 5,
children	: 2 ,
$TIAGV_{local}$:	19.6 mg/m³.

Fluoro(trimethyl)silane

Identification, physical properties

Name of substance, abbreviation:
→ Fluoro(trimethyl)silane (FTMS)
→ trimethylsilylfluoride
CAS-No.: 420-56-4
Molecular weight: 92.19 g/mol

Table A2: Physical chemical data

Melting point	-74 °C[a)]
Boiling point	16…18 °C[a)]
Water solubility	1.73 g/L at 25 °C[b)]
Vapor pressure	300 kPa at 25 °C[b)]
Log Pow	2.17 at 20 °C[b)]

[a)]: CHEMSPIDER, 2017
[b)]: U.S. EPA, 2017

Conversion factors air-concentration:
1 ppm = 3.77 mg/m³;
1 mg/m³ = 0.265 ppm

Structure:

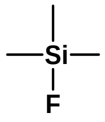

Adsorption, distribution, metabolism, elimination

There are no data available on this substance. FTMS undergoes rapid hydrolysis in

aqueous solutions, and the equilibrium constant is about 10^{-3} (Gibson and Janzen, 1972).

$$(CH_3)_3Si\text{-}F + H_2O = (CH_3)_3Si\text{-}OH + HF$$

After inhalation of trace concentrations in the air, the concentration of FTMS in tissue is expected to be very low as compared to water, which can be set as constant = 55.56 mol/L. That means, initially about 50 % of the FTMS is hydrolyzed to trimethylsilanol (TMSOH) and HF; the fluoride ion will react rapidly with calcium ions in biological tissues, so the equilibrium is expected to be shifted quantitatively to the right hand side. This assumption is consistent with the observation that FTMS is an irritant (see below). As a result, in absence of repeated dose toxicity data for FTMS, a Tentative Indoor Air Guidance Value will be based on its hydrolysis products TMSOH and HF.

TMSOH and FTMS are compared to each other in the PBTK modelling IndusChemFate v2.0 (Jongeneelen and ten Berge, 2011). Physical chemical data used are listed in Table A3.

Table A3: Physical chemical data for trimethylsilanol (TMSOH) and fluoro(trimethyl)silane (FTMS)

	**TMSOH**	**FTMS**
Molar mass	90 g/mol	92 g/mol
Density	0.814	~ 0.8
Water solubility	995 mg/L	1729 mg/L
Vapour pressure / 25 °C	2900 Pa	300000 Pa
log Kow	1.62	2.17

The results of the PBTK modelling are given in Table A4.

Based on these calculations, concentrations in tissues known to be targets for solvents and VOCs do not require an additional safety factor for FTMS if read across is done from TMSOH.

Short-term toxicity

FTMS is classified as irritating to skin, eyes and the respiratory tract (ECHA Fluoro(trimethyl)silane, 2017).

Repeated dose toxicity

There are no data available for FTMS. Repeated dose toxicity is read across from the hydrolysis products TMSOH and HF. For the hydrolysis product TMSOH, see above.

In a subchronic inhalation study rats were exposed against 0, 0.1, 1.0 or 10 ppm HF for 6 h/d and 5 d/w (DFG Hydrogen fluoride, 2001). 1.0 ppm was the NOAEC for local and systemic effects. At 10 ppm, between others, irritation of the respiratory tract, decreased body weights, altered hematology, increased relative weights of several organs and dental changes were observed.

1.0 ppm was the NOAEC for local effects in repeated exposure studies with volunteers (DFG, 2001a).

Genetic toxicity

There are no data available for FTMS. Concerning the hydrolysis products, TMSOH is not a genotoxic agent (see above). HF was negative in the bacterial reverse mutation assay, and fluorides were equivocal in *in vitro* tests, but negative in *in vivo* test (DFG Hydrogen fluoride, 2001).

Carcinogenicity

No data are available for FTMS. Concerning the hydrolysis products, TMSOH is not likely to pose a significant carcinogenic risk (see above); fluorides were positive in a carcinogenicity assay only at very high dosages which are very unlikely to occur under realistic exposure conditions (DFG, 2001a).

Reproduction toxicity

There are no data available for FTMS. As for the hydrolysis products, TMSOH is not expected to affect reproduction (see above). HF and fluorides do not affect fertility and development at dosages that do not cause general toxicity (DFG, 2006).

EXCLI Journal 2018;17:784-791 – ISSN 1611-2156
Received: June 18, 2018, accepted: August 02, 2018, published: August 07, 2018

Table A4: Calculated tissue concentrations [µmol/L] for TMSOH and FTMS

Substance [air concentration]	Venus blood	Liver	Kidney	Brain	Bone marrow
TMSOH [1 mg/m³]	0.0589	0.0669	0.0744	0.099	0.099
TMSF [1 mg/m³]	0.00277	0.00485	0.00485	0.00679	0.00679

Indoor Air Guidance Value

Fluorotrimethylsilane (FTMS) has a very limited toxicological database, but due to its chemical properties it is expected to hydrolyse rapidly in biological tissues. On hydrolysis, trimthylsilanol (TMSOH) and hydrogen fluoride are formed. Tentative indoor air guidance values were derived for TMSOH for local and systemic effects (see TMSOH).

$TIAGV_{systemic,\ TMSOH}$: 195 µg/m³ = 0.053 ppm
$TIAGV_{local,\ TMSOH}$: 19600 µg/m³ = 5.23 ppm.

For hydrogen fluoride, the evaluation of the MAK commission of the Deutsche Forschungsgemeinschaft will be used for the derivation of an Indoor Air Guidance Values (IAGV) (DFG, 2001a). For workers, the occupational exposure limit (OEL) was 2 ppm for local and 1 ppm for systemic effects (DFG, 2001a). A factor (divisor) of 2 will be introduced to cover potential higher vulnerability of the general population, and 24 h / 8 h and 7 d / 5 d to address the extended exposure periods (in total a divisor of 4.2). A further factor of 2 covers potential higher sensitivity of children. The results for HF, therefore, are

$OEL_{HF,\ local}$	2 ppm,
worker to consumer	: 2,
children:	: 2,
time scaling:	: 168/40,
$IAGV_{HF,\ local}$	0.12 ppm.

$OEL_{HF,systemic}$	1 ppm,
worker to consumer	:2,
children	:2,
time scaling	:168/40,
$IAGV_{HF,systemic}$	0.06 ppm.

Every molecule FTMS hydrolyses to one molecule TMSOH and one molecule HF. The tentative indoor air guidance value for FTMS, therefore, is calculated as the result of the two hydrolysis products:

$$1 = \frac{TIAGV_{FTMS}}{IAGV_{HF}} + \frac{TIAGV_{FTMS}}{TIAGV_{TMSOH}} \Leftrightarrow$$

$$\frac{1}{TIAGV_{FTMS}} = \frac{1}{IAGV_{HF}} + \frac{1}{TIAGV_{TMSOH}}$$

$TIAGV_{FTMS,\ local}$ = 0.12 ppm = 442 µg/m³
$TIAGV_{FTMS,systemic}$ = 0.028 ppm = 107 µg/m³

1-Chloro-2-propanol

Industrial chloropropanol (CP) is a 2:1 mixture of 1-chloro-2-propanol and 2-chloro-1-propanol.

Identification, physical properties

Name of substance, abbreviation:
→ 1-chloro-2-propanol
→ 2-chloro-1-propanol (CP);
CAS-No.: 127-00-4; 19210-21-0 and 78-89-7
Molecular weight: 94.54 g/mol

Table A5: Physical chemical data

Melting point	-55 °C[a)]
Boiling point	127 °C[b)]
Water solubility	140 g/L at 25 °C[a)]
Vapor pressure	650 Pa at 20 °C[b)]
Log Pow	0.56 at 20 °C[a)]

[a)]: U.S. EPA, 2017
[b)]: GESTIS, 2017a

Conversion factors air-concentration:
1 ppm = 3.87 mg/m³;
1 mg/m³ = 0.259 ppm

EXCLI Journal 2018;17:784-805 – ISSN 1611-2156
Received: June 18, 2018, accepted: August 02, 2018, published: August 07, 2018

Structure:

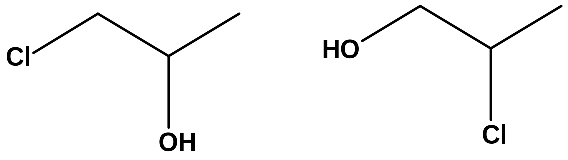

CAS-No. 127-00-4; 19210-21-0 and 78-89-7

Adsorption, distribution, metabolism, elimination

After subcutaneous dosing, rats excreted 2-hydroxypropyl mercapturic acid in the urine (Barnsley, 1966).

Following inhalation exposure to 1-chloro-2-propanol, half-lives for the excretion of ^{14}C in urine and $^{14}CO_2$ in exhaled air were 3 and 7 h in the rat (Bond et al., 1988). Directly after exposure, highest concentrations were found in kidneys, livers, trachea and nasal turbinate. Elimination from tissue was biphasic with half lives of 1…3 h and 40…80 h.

Short-term toxicity

Data for short term toxicity are given in the documentation of the ACGIH (2002).

LD_{50}/oral/rat:	243…300 mg/kg
LD_{50}/oral/guinea pig:	720 mg/kg
LD_{50}/oral/mouse:	580 mg/kg
LD_{50}/oral/dog:	>100, < 250 mg/kg
LD_{50}/dermal/rabbit:	440 mg/kg
LC_{50}/4 h/rat:	1000 ppm

CP is not irritating to the skin but a severe irritant to the eyes of rabbits.

Repeated dose toxicity

In a subacute inhalation study with rats, 30 ppm for 6 h/d over 14 d was the NOAEC; 100 ppm caused congestion in the lungs and perivascular edema (ACGIH, 2002).

In drinking water studies with mice and rats, pancreas, kidneys and livers were the target organs. In the subchronic study, rats and mice received 0, 33, 100, 330, 1,000, or 3,300 ppm in drinking water (5, 10, 35, 100, or 220 mg/kg/d). At 100, 1000 and 3300 ppm, but not at 330 ppm, male rats showed cytoplasmatic vacuolization in the liver. At 3300 ppm, body weights were decreased and males had significantly reduced epididymis weights and increased incidence of abnormal sperm. 1000 ppm can be regarded as NOAEL. In mice, 330 ppm was the NOAEL, and higher dosages caused increased liver and kidney weights, and renal tubulus vacuolization in males.

In a chronic drinking water study, rats received 0, 75, 160 or 325 ppm, mice received 0, 125, 250 or 500 ppm CP (ACGIH, 2002; NTP, 1998). No treatment-related neoplasms or pre-neoplastic lesions were observed in this study.

Genetic toxicity

CP was genotoxic in *S. typhimurium* strains TA 100 and TA 1535, in the mouse lymphoma assay, and induced chromosomal damage in CHO cells *in vitro* and in rat bone marrow *in vivo* after oral dosing. An *in vivo* micronucleus test with oral administration to mice was negative (ACGIH, 2002).

Carcinogenicity

During a chronic drinking water study, rats received 0, 75, 160 or 325 ppm, mice received 0, 125, 250 or 500 ppm CP. No treatment-related neoplasms or pre-neoplastic lesions were observed in this study (NTP, 1998).

Reproduction toxicity

In a one-generation drinking water study, 650 and 1300 ppm caused significantly reduced body weights in dams; fertility and development were not affected. 300 ppm was the NOAEL (15 mg/kg/d) (ACGIH, 2002).

Indoor Air Guidance Value

Chloropropanol is genotoxic, but did not induce significant increase in tumors in drinking water studies with rats and mice (NTP, 1998). In a one generation study in rats, CP did not affect fertility and development (ACGIH 2002); for that reason, the factor to cover children is set to 1. In a subacute inhalation study with rats, a NOAEC of 30 ppm (116 mg/m³) could be established (ACGIH, 2002). Based on available data, the ACGIH (2002)

derived an occupational exposure limit of 1 ppm (3.78 mg/m³). The NOAEC of the subacute rat study will be taken as PoD for the derivation of the Indoor Air Guidance Value (IAGV):

$NOAEC_{subacute}$ 30 ppm,
subacute to chronic : 6,
time scaling : 24/6,
inter-species differences : 2,5,
intra-species differences : 10,
children : 1 ,
$IAGV_{CP}$ 0.05 ppm = 195 µg/m³.

If the OEL of 1 ppm was taken as point of departure, factors (divisors) of 2 for worker to consumer, 4.2 for 24 h/d and 7 d/w exposure, and a factor of 2 for children would result in an IAGV of 0.06 ppm.

2,2,3,3-Tetramethyl-succinodinitrile

Identification, physical properties

Name of substance, abbreviation:
→ Tetramethylsuccinodinitril, TMSD;
→ 2,3-dicyano-2,3-dimethylbutane.
CAS-No.: 3333-52-6
Molecular weight: 136.19 g/mol

Table A6: Physical chemical data

Melting point	171 °C[a)]
Boiling point	---
Water solubility	5.32 g/L at 25 °C[b)]
Vapor pressure	5 Pa at 25 °C[b)]
Log Pow	1.11 at 20 °C[b)]

a): DFG, 2001b
b): U.S. EPA, 2017

Conversion factors air-concentration:
1 ppm = 5.6 mg/m³;
1 mg/m³ = 0.18 ppm

Structure:

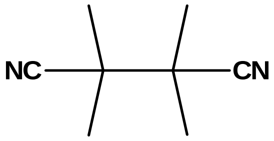

Adsorption, distribution, metabolism, elimination

Other than succinodinitrile, TMSD does not release cyanide ions when given to mice; this was proven by the absence of thiocyanate formation and the fact, that liver deactivation by carbon tetrachloride reduced the toxicity of succinodinitrile, but not that of TMSD (Doherty et al., 1982). Acute toxicity data indicate a complete resorption by oral administration and similar toxicity by all routes of exposure.

Short-term toxicity

Acute toxicity data are summarized in the MAK value documentation for TMSD (DFG, 2001b).

LD_{50}/oral/rat: 25…50 mg/kg;
LD_{50}/oral/rabbit: 17.5 mg/kg;
LD_{50}/oral/guinea pig: 17.5…25 mg/kg;
LD_{50}/dermal/rabbit/24 h: 79…316 mg/kg;
LC_{50}/rat/6 h: about 80 mg/m³
LD_{50}/mouse/i.p.: 18 mg/kg
LD_{50}/rat/i.p.: 18 mg/kg
LD_{50}/mouse/i.v. 18 mg/kg

Symptoms of acute intoxication are tremor, convulsions and impaired breathing which indicate neurotoxicity.

TMSD is not irritating to the skin and slightly irritating to the eyes.

Repeated dose toxicity

Inhalation exposure of rats against 19 mg/m³ for 6 h/d over 10 days caused impaired breathing. Body weight gain was not depressed, liver, kidney, brain and the spine were not affected. The lungs showed indications of pneumonitis (DFG, 2001b).

In a subchronic gavage study rats received daily doses of 1, 3 or 10 mg/kg TMSD (DFG, 2001b; Johannsen and Levinskas, 1986). In female rats, 3 mg/kg caused a significant decrease in body weight gain. For male rats, all doses resulted in increased kidney weight, proximal tubulus degeneration and hyaline droplet formation. These kidney effects are regarded as species- and gender specific. Therefore, the NOAEL is 1 mg/kg.

Four female and male beagles received daily doses of 0, 0.3, 1.0 or 3.0 mg/kg TMSD for 90 days (Johannsen and Levinskas, 1986). At the top dose, liver weights were increased and body weight gain was depressed. At 1 mg/kg, there was only a slight depression in body weight gain in female dogs. Behavior, gross pathology and histopathology as well as hematology, clinical chemistry and urine analysis did not show adverse effects. At the top dose, thiocyanate was detectable in blood. The NOAEL is 1 mg/kg.

Workers exposed against TMSD reported headache, startle reactions, dizziness, unconsciousness and convulsions. It is not clear whether or not TMSD was the only potential source for these health effects (Reinl, 1957).

In a more recent study, headache, dizziness, convulsions and hypoglycemia were correlated with workplace exposure against TMSD in the PVC foam industry. Symptoms disappeared as soon as the previously exceeded Swiss occupational exposure limit of 3 mg/m³ was met (about 0.4…0.5 mg/kg/d) (Ensslin and Kofler, 2014).

Genetic toxicity

TMSD was negative in the bacterial reverse mutation assay and the mouse lymphoma assay with and without metabolic activation (Seifried et al., 2006).

Carcinogenicity

A cancer study was not identified. Based on the data of the mutagenicity tests, TMSD is not expected to have a noticeable carcinogenic potential.

Reproduction toxicity

Doherty et al. (1983) reported the reproduction toxicity effects of TMSD on hamster. A limited teratogenicity study was performed with pregnant hamsters receiving a single intra-peritoneal dose of 0, 4.9, 9.9 or 20 mg/kg BW on gestation day 8. The high dose caused exencephaly. At lower doses there was no gross malformation observable. The mid and high dose caused a significant decreased fetal body size. Some dams of the mid dose group and all animals of the high dose group showed hyperstartle reactions and convulsions. Teratogenic effects are regarded as secondary to parental toxicity. Dams receiving the anticonvulsant Trimethadione (Ca-channel blocker) before TMSD administration were less severely affected.

There are no studies concerning potential effects on fertility and post-partum development. Due to lack of histopathological changes in female and male gonads in the subchronic study, the likelihood of adverse effects on fertility is regarded as being of low concern.

Indoor Air Guidance Value

Tetraemethylsuccinodinitrile (TMSD) was negative in *in vitro* mutagenicity tests. Data concerning effects on development are very limited (Doherty et al., 1983), and concerning fertility it is not clear whether or not histopathology of gonads was performed in subchronic studies (Johannsen and Levinskas, 1986). In sub-chronic oral studies with rats and dogs, the NOAEL was 1 mg/kg b.w., which serves as a starting point; in both species, 3 mg/kg was the LOAEC (DFG, 2001b). Both subchronic studies, as well as acute toxicity studies indicate that inter-species differences are very small to negligible. For that reason, a divisor of 1.4 is introduced for the allometric scaling from dog to man. Differences in toxicodynamics are regarded as negligible. For intra-species extrapolation, a factor of 10 is used for the consumer. For subchronic to chronic exposure, the factor applied is 2, and a factor 2 addresses children as vulnerable population. Acute toxicity data have demonstrated that orally applied TMSD is completely resorbed; therefore, the oral to inhalation extrapolation factor is 1.

$NOAEC_{systemic}$:	1 mg/kg BW,
inter-species (dog -> man)	: 1.4,
remaining inter-species	: 1,
s.c. to chronic exposure:	: 2,
intraspecies differences:	: 10,
children	: 2,
oral to inhalation	: 1,

EXCLI Journal 2018;17:784-805 – ISSN 1611-2156
Received: June 18, 2018, accepted: August 02, 2018, published: August 07, 2018

and for a person with 60 kg body weight, 20 m^3 breathing volume per day
TIAGV: 54 µg/m^3.

The teratogenicity study with parenteral dosing is regarded as not relevant for indoor air inhalation exposure; however, the potential for reproduction toxicity needs to be clarified. Because of this, the indoor air guidance value is tentative.

Triethylenediamine

For 1,4-diazabicyclo[2.2.2]octane, (triethylenediamine) the REACH registration dossier provides data on a subacute inhalation study and a combined subacute / reproduction toxicity screening study after gavage administration in rats (ECHA Triethylendiamine, 2017).

Identification, physical properties

Data are retrievable from the European Chemicals Agency website (ECHA Triethylendiamine, 2017).

Name of substance, abbreviation:
→ 1,4-Diazabicyclo[2.2.2]octane,
→ Triethylenediamine.
CAS-No.: 280-57-9
Molecular weight: 112.17 g/mol

Table A7: Physical chemical data

Melting point	158 °C
Boiling point	173 °C
Water solubility	610 g/L at 25 °C
Vapor pressure	43 Pa at 23 °C[b)]
Log Pow	-0.47 at 20 °C[a)]

Conversion factors air-concentration:
1 ppm = 4.6 mg/m^3;
1 mg/m^3 = 0.218 ppm

Structure:

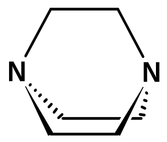

Adsorption, distribution, metabolism, elimination

There are no data available on this substance.

Short-term toxicity

Data are listed in the registration dossier (ECHA Triethylendiamine, 2017).
LD_{50}/oral/rat: 700 mg/kg;
LD_{50}/dermal/rabbit/24 h: >3200 mg/kg;
LC_0/rat/8 h 1900 mg/m^3.
LD_{50}/mouse/i.p.: 400 mg/kg.

Triethylenediamine causes severe eye irritation with irreversible effects; the substance is irritating to the skin, it is not skin sensitizing in the guinea pig.

Repeated dose toxicity

In a combined reproduction toxicity / subacute toxicity gavage study, rats received 0, 100, 300 or 1000 mg/kg triethylenediamine dissolved in water. Urine analysis and ophthalmology were not performed. At 1000 mg/kg, weight gain was reduced in males and females, and females showed increased liver weights. Inflammatory changes in the kidney were observed in mid- and top-dose males and the kidneys and urinary bladders of top dose females. 100 mg/kg was identified as NOAEL (ECHA Triethylendiamine, 2017).

5 male and 5 female rats per dose were exposed nose only against 0, 5.8, 63 or 620 mg/m^3 triethylendiamine as aqueous aerosol for 6 h/d, 5 d/w over 28 days. In the high dose group, one female died; all animals showed signs of severe inflammations of the eyes, ears and noses; ophthalmology revealed corneal damage in all high dose animals. In the high dose, hematology showed increased urea levels in both genders. In the high dose, males had a reduced food intake and slightly increased serum aspartate aminotransferase without histopathological correlate. Adrenals weight was increased in the high dose group males without histopathological correlate, and testicle weights were increased in the mid and high dose group. Microscopically laryngitis was observed dose-dependently in the

EXCLI Journal 2018;17:784-805 – ISSN 1611-2156
Received: June 18, 2018, accepted: August 02, 2018, published: August 07, 2018

mid and high dose group. For local effects, the NOAEC was 5.8 mg/m³; the registrant regards 620 mg/m³ as NOAEC for systemic toxicity. Urine analysis was not included (ECHA Triethylendiamine, 2017; BG Chemie, 1995). Recalculated as daily dose, 620 mg/m³ for 6 h/d is about 150 mg/kg/d which is in between the NOAEL and the LOAEL of the gavage study.

Genotic toxicity

Triethylendiamine was not mutagenic in the bacterial reverse mutation assay with and without metabolic activation. Triethylendiamine did not induce micronuclei in rat bone marrow after oral administration. A dose-dependent decrease in PCE/NCE ratio was observable (ECHA Triethylendiamine, 2017; BG Chemie, 1995). Based on these data, triethylendiamine is unlikely to pose a critical genotoxic risk.

Carcinogenicity

A cancer study was not identified. Based on the data of the mutagenicity tests, triethylendiamine is not expected to have a critical carcinogenic potential.

Reproduction toxicity

In a combined reproduction toxicity / subacute toxicity gavage study, rats received 0, 100, 300 or 1000 mg/kg triethylendiamine dissolved in water. For the F0 generation, the NOAEL was 100 mg/kg (see above). The top dose caused reduced litter size. At 300 mg/kg, litter size, pup survival and fertility parameters were not affected, and this dose is the NOAEL for the F1 generation (ECHA Triethylendiamine, 2017).

In a teratogenicity study, pregnant rats received 0, 3.3 or 33 mg/kg triethylendiamine per day during gestation days 1 to 19 (BG Chemie, 1995). Adverse effects were not observable.

Indoor Air Guidance Value

Triethylenediamine was registered under Regulation (EU) 1907/2006 (REACH), but the registrant did not derive a DNEL for inhalation exposure of the consumer (ECHA Triethylendiamine, 2017). The registrant regards 620 mg/m³ to be the systemic NOAEC in the subacute inhalation study. However, at this concentration, absolute and relative testicle weights, adrenal weights, blood urea and aspartate amino transferase were elevated, and food intake was reduced in male rats. Histopathology did not reveal any adverse effects at the top concentration, but urine analysis was not performed, and tests concerning fertility effects on male rats after inhalation exposure are missing. After oral exposure, triethylendiamine did affect neither fertility nor development in a screening study with rats (ECHA Triethylendiamine, 2017). As a precautionary measure, 63 mg/m³ are regarded as the appropriate point of departure for the derivation of a tentative Indoor Air Guidance Value.

For systemic effects, the NOAEC from the inhalation study is extrapolated by several divisors:

$NOAEC_{rat, systemic}$	63 mg/m³,
subacute to chronic	: 6,
6 h/d to 24 h/d	: 4,
5 d/w to 7 d/w	: 7/5,
Inter-species	: 2.5,
Intra-species	: 10,
children	: 2,
$TIAGV_{systemic}$	38 µg/m³.

If the NOAEL of the reproduction toxicity screening study is taken as point of departure, against the endpoint developmental toxicity and fertility a factor of 5 needs to be introduced due to limited sensitivity of screening studies. The corrected NOAEL would than be 60 mg/kg/d. A factor of 10 for each, inter- and intra-species extrapolation would result in a $NOAEL_{man}$ of 0.6 mg/kg/d. For a person of 60 kg body weight and 20 m³ breathing volume per day, with a correction factor of 2 (divisor) for oral to inhalation extrapolation results in

$TIAGV_{repro}$ 900 µg/m³.

Concerning local effects, the $NOAEC_{local}$ of the subacute inhalation study is the starting point.

EXCLI Journal 2018;17:784-805 – ISSN 1611-2156
Received: June 18, 2018, accepted: August 02, 2018, published: August 07, 2018

$NOAEC_{rat, local}$	5.8 mg/m³,
subacute to chronic	6,
6 h/d to 24 h/d	4,
5 d/w to 7 d/w	7/5,
Inter-species	2.5,
Intra-species	5,
children	2,
$TIAGV_{local}$	7 µg/m³.

Propanal (Propionaldehyde)

Identification, physical properties

Name of substance, abbreviation:

→ propionaldehyde;

→ propanal;

CAS-No.: 123-38-6

Molecular weight: 58.08 g/mol

Table A8: Physical chemical data

Melting point	-81 °C
Boiling point	49 °C
Water solubility	miscible
Vapor pressure	341 kPa at 20 °C
Log Pow	3.43 at 20 °)

Conversion factors air-concentration:
1 ppm = 2.37 mg/m³;
1 mg/m³ = 0.421 ppm

Structure:

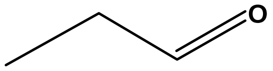

Data are taken from the GESTIS database (GESTIS, 2017b).

Adsorption, distribution, metabolism, elimination

Human liver aldehyde dehydrogenase oxidizes propanal at a similar rate as acetaldehyde *in vitro*. V_{max} = 6.53 µmol NADH/min/mg protein, K_M = 808 µM for rat liver microsomes; anaesthetized dogs retained up to 80 % of inhaled propionaldehyde (ECHA Propanal, 2017).

.

Short-term toxicity

LD_{50}/oral/rat:	1690 mg/kg
LDC_{50}/dermal/rabbit:	2460 mg/kg
LC_{50}/4 h/rat:	> 4600 mg/m³

Propanal is a strong eye irritant which causes irreversible effects; it is not irritating to the skin of rabbits and not a skin sensitizer in the LLNA (ECHA Propanal, 2017).

In the acute respiratory irritation test in rats, propanal had a 3-h RD_{50} of about 6800 ppm, about twice the value of acetaldehyde; in mice, the picture was reversed with an RD_{50} of 2052 ppm for propanal and 2845 ppm for acetaldehyde (Babiuk et al., 1985).

Repeated dose toxicity

In a subacute inhalation study, rats were exposed for 6 h/d and 7 d/w against 0, 150, 750 and 1500 ppm (362, 1810, 3620 mg/m³) propanal. In the high dose group, partly in the middle dose group food intake and body weight was reduced in female rats; male rats had significantly increased kidney weights; vacuolization of the olfactoric epithelium occurred in all dose groups; in high dose groups atrophy of the olfactoric epithelium was observable. The registrant concludes a NOEC of 150 ppm for systemic toxicity; concerning irritation of the olfactoric epithelium, 150 ppm is the LOEC (ECHA Propanal, 2017).

Genetic toxicity

Propanal was negative in the bacterial reverse mutation assay with and without metabolic activation, did not induce sister chromatid exchanges (SCE) in human lymphocytes and did not induce unscheduled DNA synthesis in rat and human hepatocytes; however, it induced point mutations in the HGPRT test, was positive in the alkaline elution assay with CHO cells and in the *in vitro* chromosomal aberration test, and caused SCE in V79 cells and DNA-protein cross-links (ECHA Propanal, 2017).

In the micronucleus test in mice after intraperitoneal administration, in males there was a significant increase in micronuclei at the top dose; PCE were reduced in the high dose group; however, because of the absence

of a dose-response, and because the control group of male mice had a very low incidence of micronuclei, the registrant concluded propanal to be negative in this test (ECHA Propanal, 2017).

Aldehyde dehydrogenase can detoxify propanal to propionic acid; however, a drop in pH in conjunction with this reaction may trigger secondary effects. In rat lungs, aldehyde dehydrogenase has 15 % activity compared to the liver (Yoon et al., 2005). Therefore, mutagenicity tests targeting the respiratory tract might be considered.

Carcinogenicity

No studies identified.

Reproduction toxicity

In a reproduction toxicity screening study, rats were exposed for 6 h/d and 7 d/w against 0, 150, 750 and 1500 ppm propionaldehyde. Fertility and development were not affected by the exposure (ECHA Propanal, 2017).

Indoor Air Guidance Value

For propanal, some *in vitro* tests indicate genotoxicity whereas others were negative. The sole *in vivo* study identified indicates some weak activity in bone marrow of male mice. For acetaldehyde, the MAK-Commission of the Deutsche Forschungsgemeinschaft has drawn the conclusion that it is genotoxic, but irritation is expected to be a requisite for tumor development in chronically exposed rats; therefore, 50 ppm, the subacute NOAEC from rat inhalation studies, divided by a factor of 3, is proposed as MAK-value (DFG, 2013). 150 ppm are a LOEC concerning vacuolization of the olfactoric epithelium for propanal. Irritating potencies are comparable between propanal and acetaldehyde. 150 ppm will be taken as a NOAEC for systemic, and as LOEC for local toxicity. The genotoxic potential of propanal needs further clarification. Concerning reproduction toxicity, data from a rat screening study with inhalation exposure are available (ECHA Propanal, 2017).

$NOAEC_{subacute}$ 150 ppm,
Inter-species : 2.5,
Intra-species : 10,
children : 2,
subacute to chronic: : 6,
time scaling : 24 h / 6 h,
$IAGV_{propanal,systemic}$ 0.12 ppm = 300 µg/m³.

For local effects, the LOEC is to be divided by a factor of 3, but for intra-species differences a factor of 5 instead of 10 is used.
$IAGV_{propanal,local}$ 200 µg/m³.

For reproduction toxicity, the value is
$NOAEC_{subacute}$ 1500 ppm,
Inter-species : 2.5,
Intra-species : 10,
screening study: : 5,
time scaling : 24 h / 6 h,
$TIAGV_{propanal,repro}$ 3 ppm = 7.11 mg/m³.

REFERENCFES

ACGIH. American Conference of Governmental Industrial Hygienists. 1-Chloro-2-propanol and 2-Chloro-1-propanol. 2002. http://www.acgih.org/forms/store/ProductFormPublic/1-chloro-2-propanol-and-2-chloro-1-propanol-tlv-r-chemical-substances-7th-edition-documentation; accessed on 12 june 2018.

Babiuk C, Steinhagen WH, Barrow CS. Sensory irritation response to inhaled aldehydes after formaldehyde pretreatment. Toxicol Appl Pharmacol. 1985;79:143-9.

Barnsley EA. The formation of 2-hydroxypropylmercapturic acid from 1-halogenopropanes in the rat. Biochem J. 1966;100:362-72.

BG Chemie, Berufsgenossenschaft Rohstoffe und Chemische Industrie. Toxikologische Bewertung Nr. 69: Triethylendiamin. 06/1995. Summary available at: https://www.bgrci.de/fileadmin/BGRCI/Downloads/DL_Praevention/Fachwissen/Gefahrstoffe/TOXIKOLOGISCHE_BEWERTUNGEN/Bewertungen/ToxBew069-K.pdf.

Bond JA, Birnbaum LS, Dahl AR, Medinsky MA, Sabourin PJ, Henderson RF. Disposition of inhaled 1-chloro-2-propanol in F344/N rats. Toxicol Appl Pharmacol. 1988;95:444-55.

CHEMSPIDER. http://www.chemspider.com/Chemical-Structure.9485.html?rid=9d0773e7-7572-4efd-a99e-ecbf0197135e; accessed on 21 june 2017.

DFG, Deutsche Forschungsgemeinschaft. MAK value documentation for hydrogen fluoride. 2001a. http://onlinelibrary.wiley.com/doi/10.1002/3527600418.mb766439e3214/pdf; accessed on 21 june 2017.

DFG, Deutsche Forschungsgemeinschaft. MAK value documentation for tetramethylsuccinnitril. 2001b. https://onlinelibrary.wiley.com/doi/pdf/10.1002/3527600418.mb333352d0033; accessed on 30 may 2017.

DFG, Deutsche Forschungsgemeinschaft. MAK value documentation for hydrogen fluoride. 2006. http://onlinelibrary.wiley.com/doi/10.1002/3527600418.mb766439e4014/pdf; accessed on 21 june 2017.

DFG, Deutsche Forschungsgemeinschaft. MAK value documentation for acetaldehyde. 2013. http://onlinelibrary.wiley.com/doi/10.1002/3527600418.mb7507e4413/pdf; accessed on 26 june 2017.

Doherty PA, Smith RP, Ferm VH. Tetramethyl substitution on succinonitrile confers pentylenetetrazol-like activity and blocks cyanide release in mice. Pharmacol Exp Ther. 1982;223:635-41.

Doherty PA, Smith RP, Ferm VH. Comparison of the teratogenic potential of two aliphatic nitriles in hamsters: succinonitrile and tetramethylsuccinonitrile. Fund Appl Toxicol. 1983;3:41-8.

ECHA Fluoro(trimethyl)silane. 2017. https://echa.europa.eu/de/information-on-chemicals/cl-inventory-database/-/discli/details/126849; accessed on 21 june 2017.

ECHA Hexamethyldisiloxane. 2017. https://echa.europa.eu/de/registration-dossier/-/registered-dossier/15156; accessed on 20 june 2017.

ECHA Hydroxy(trimethyl)silane. 2017. https://echa.europa.eu/de/substance-information/-/substanceinfo/100.012.650; accessed on 20 june 2017.

ECHA Propanal. 2017. https://echa.europa.eu/de/registration-dossier/-/registered-dossier/13148; accessed on 26 june 2017.

ECHA Triethylendiamine. 2017. https://echa.europa.eu/de/registration-dossier/-/registered-dossier/13591; accessed at 31 may 2017.

Ensslin AS, Kofler MF. Convulsions and hypoglycaemia due to tetramethyl succinonitrile intoxication in the polyvinylchloride (PVC) industry: a 4-year follow-up. Int J Hyg Environ Health. 2014;27:188-95.

GESTIS. 1-Chlor-2-propanol. 2017a. http://gestis.itrust.de/nxt/gateway.dll/gestis_de/000000.xml?f=templates$fn=default.htm$vid=gestisdeu:sdbdeu$3.0 ; accessed on 21 june 2017.

GESTIS. Propionaldehyd. 2017b. http://gestis.itrust.de/nxt/gateway.dll/gestis_de/000000.xml?f=templates$fn=default.htm$vid=gestisdeu:sdbdeu$3.0; accessed on 26 june 2017.

GESTIS. Trimethylsilanol. 2017c. http://gestis.itrust.de/nxt/gateway.dll/gestis_de/000000.xml?f=templates$fn=default.htm$vid=gestisdeu:sdbdeu$3.0 ; accessed at 20 june 2017.

Gibson JA, Janzen AF. N.M.R. study of base-catalyzed hydrolysis of trimethylfluorosilane. Can J Chem. 1972;50:3087-92.

Isquith A, Matheson D, Slesinski R. Genotoxicity studies on selected organosilicon compounds: in vitro assays. Food Chem Toxicol. 1988a;26:255-61.

Isquith A, Matheson D, Slesinski R. Genotoxicity studies on selected organosilicon compounds: in vivo assays. Food Chem Toxicol. 1988b;26:263-6.

Johannsen FR, Levinskas GJ. Subchronic toxicity of tetramethylsuccinonitrile. Fund Appl Toxicol. 1986:7:41-8.

Jongeneelen FJ, ten Berge W. A generic, cross-chemiccal predictive PBTK model with multiple entry routes running as application in MS Excel; design of the model and comparison of predictions with experimental results. Ann Occup Hyg. 2011;55:841-64. See also: http://cefic-lri.org/toolbox/induschemfate/.

NIHS, National Institute of Health Sciences, Japan. Trimethylsilanol. http://dra4.nihs.go.jp/mhlw_data/home/file/file1066-40-6.html; accessed on 20 june 2017.

NTP. Technical report on the toxicology and carcinogenesis studies of 1-chloro-2-propanol (technical grade) in F344/N rats and B6C3F1 mice. NTP TR 477; NIH publication-no. 98-3967. 1998. https://ntp.niehs.nih.gov/testing/status/agents/ts-127004.html; accessed on 17 june 2017.

Reinl W. Erkrankungen durch Tetramethylbernsteinsaeuredinitril bei der Schaumstoffherstellung. Arch Toxicol. 1957:16:367-80.

Rutz W, Lange D, Kelling H. Kondensation von Trimethylsilanol. Z Anorg Allgem Chem. 1985;528:98-106.

Sefcik J, Rankin SE, Kirchner SJ, McCormick AV. Esterification, condensation and deprotonation equilibria of trimethylsilanol. J Non-crystall Solids. 1999;258:187-97.

Seifried HE, Seifried RM, Clarke JJ, Junghans TB, San RHC. Compilation of Two decades of mutagenicity test results with the Ames Salmonella typhimurium and L5178Y mouse lymphoma cell mutation assays. Chem Res Toxicol. 2006;19:627-44.

Sigma-Aldrich. Sigma-Aldrich Safety Data Sheet Trimethylsilanol, version 5.1, 2014. https://www.sigmaaldrich.com/MSDS/MSDS/DisplayMSDSPage.do?country=DE&language=en&productNumber=725986&brand=ALDRICH&PageToGoToURL=https%3A%2F%2Fwww.sigmaaldrich.com%2Fcatalog%2Fsearch%3Fterm%3D1066-40-6%26interface%3DCAS%2520No.%26N%3D0%26mode%3Dpartialmax%26lang%3Den%26region%3DDE%26focus%3Dproduct; Accessed on 20 june 2017.

U.S. EPA, United States Environmental Protection Agency. EPISUITE v.4.1. https://www.epa.gov/tsca-screening-tools/download-epi-suitetm-estimation-program-interface-v411. Accessed on 15 may 2017.

Yoon M, Madden MC, Barton HA. Comparison of aldehyde dehydrogenase (ALDH) activity in the liver and lung. Toxicol Sci. 2005;84(1-S):392.
